# Exosomal microRNA‐16‐5p from human urine‐derived stem cells ameliorates diabetic nephropathy through protection of podocyte

**DOI:** 10.1111/jcmm.14558

**Published:** 2019-09-30

**Authors:** Yu‐Rui Duan, Bao‐Ping Chen, Fang Chen, Su‐Xia Yang, Chao‐Yang Zhu, Ya‐Li Ma, Yang Li, Jun Shi

**Affiliations:** ^1^ Department of Nephrology Huaihe Hospital of Henan University Kaifeng China; ^2^ Department of Urology Huaihe Hospital of Henan University Kaifeng China

**Keywords:** diabetic nephropathy, exosomes, human urine‐derived stem cells, microRNA‐16‐5p, podocyte, vascular endothelial growth factor A

## Abstract

Diabetic nephropathy (DN) remains one of the severe complications associated with diabetes mellitus. It is worthwhile to uncover the underlying mechanisms of clinical benefits of human urine‐derived stem cells (hUSCs) in the treatment of DN. At present, the clinical benefits associated with hUSCs in the treatment of DN remains unclear. Hence, our study aims to investigate protective effect of hUSC exosome along with microRNA‐16‐5p (miR‐16‐5p) on podocytes in DN *via* vascular endothelial growth factor A (VEGFA). Initially, miR‐16‐5p was predicated to target VEGFA based on data retrieved from several bioinformatics databases. Notably, dual‐luciferase report gene assay provided further verification confirming the prediction. Moreover, our results demonstrated that high glucose (HG) stimulation could inhibit miR‐16‐5p and promote VEGFA in human podocytes (HPDCs). miR‐16‐5p in hUSCs was transferred through the exosome pathway to HG‐treated HPDCs. The viability and apoptosis rate of podocytes after HG treatment together with expression of the related factors were subsequently determined. The results indicated that miR‐16‐5p secreted by hUSCs could improve podocyte injury induced by HG. In addition, VEGA silencing could also ameliorate HG‐induced podocyte injury. Finally, hUSC exosomes containing overexpressed miR‐16‐5p were injected into diabetic rats via tail vein, followed by qualification of miR‐16‐5p and observation on the changes of podocytes, which revealed that overexpressed miR‐16‐5p in hUSCs conferred protective effects on HPDCs in diabetic rats. Taken together, the present study revealed that overexpressed miR‐16‐5p in hUSC exosomes could protect HPDCs induced by HG and suppress VEGFA expression and podocytic apoptosis, providing fresh insights for novel treatment of DN.

## 1 INTRODUCTION

Diabetic nephropathy (DN), one of the most common complications of diabetes mellitus, often results in end‐stage renal disease, such as type I diabetes, type II diabetes and other secondary subtypes of diabetes.^1^ Ageing, hyperglycaemia, obesity, hypertension, inheritance, sedentary lifestyle and smoking are considered to be the hallmark risk factors associated with the occurrence of DN.^2^ Damage to the glomerular podocytes occurs with continually high blood glucose levels, with studies highlighting hyperglycaemia as a key event occurring in the early stages of DN.^3^ The occurrence and development of DN are associated with damage of podocytes.^4^ Moreover, studies have suggested that high glucose (HG) leads to a decrease in the number of podocytes, inducing the apoptosis of cultured podocytes. HG also gives rise to proteinuria and speeds up the regression of foot processes.^5^ Furthermore, protrusion of the foot process and detachment of podocytes from the glomerular basement membrane contribute to the occurrence of proteinuria,^6^ highlighting the importance of inhibiting podocyte apoptosis for the treatment of renal diseases, including DN.^7^

Stem cells, which can be self‐renewed and differentiated into one or more pedigrees to produce specialized cell types, have been speculated as a potential therapeutic approach for DN.^8^ Interestingly, consistent with the characteristics of marrow mesenchymal stem cells (MSCs), human urine‐derived stem cells (hUSCs) isolated from human urine have been reported to secrete proangiogenic growth factors capable of maintaining the differentiation potential to endothelial cells, emphasizing their significance in the treatment of diabetic diseases.^9^, ^10^ Notably, hUSCs possess superior proliferative abilities when compared to adipose‐derived stem cells, with studies indicating the ability of hUSCs to differentiate into osteoblasts, adipocytes and chondrocytes in the presence of optimal conditions, all of which highlight the potential of hUSCs as a promising cell source for regenerative medicine and novel cell therapies.^11^

Exosomes, nanoscale vesicles secreted by a variety of cells, are involved in intracellular communication and material transport *via* directing action of signal molecules on the surface of the cell membrane and the mediation of cell content during membrane fusion.^12^ Commonly, exosomes are nano‐sized vesicles (40‐100 nm) that can be transported between cells, carrying proteins, microRNAs (miRNAs) and messenger RNAs (mRNAs).^13^ A growing body of evidence has suggested that MSC‐derived exosomes (MSC‐Exo) are capable of carrying and secreting miRNA "marker signatures," which is a key factor in the treatment of chronic inflammation and the enhancement of the therapeutic effect of MSCs.^14^ MicroRNAs are a group of small non‐coding RNA molecules associated with the initiation and development of diabetes mellitus.^15^ Intriguingly, a previous study demonstrated that circulating levels of miR‐16‐5p are highly expressed in women with gestational diabetes mellitus (GDM) when compared to controls, suggesting the potential use of miR‐16‐5p as a non‐invasive biomarker for GDM.^16^ Furthermore, miR‐16‐5p directly targets vascular endothelial growth factor A (VEGFA), which has been widely reported as a critical mediator of DN, and participates in endogenous angiogenesis of endothelial cells.^17^, ^18^ Notably, the intravenous administration of hUSCs‐Exo in streptozocin (STZ)‐induced rat models has been found to reduce urine volume and urinary microalbumin excretion as well as inhibit podocyte apoptosis and promote angiogenesis.^19^ However, as to whether miR‐16‐5p is present in hUSCs‐Exo and manipulates VEGFA to protect podocytes from DN remains largely unknown. Hence, the current study set out to investigate the role of hUSCs‐Exo combined with miR‐16‐5p in DN working in tandem with VEGFA.

## 2 MATERIALS AND METHODS

### 2.1 Ethical statements

The animal experiments in our study were strictly carried out in accordance with the instructions of animal protection and animal usage issued by American National Institutes of Health.

### 2.2 Bioinformatics

Abnormal angiogenesis is often observed in DN, with VEGFA commonly implicated in the development of the condition.^20^ VEGFA has been shown to exhibit elevated levels among rat DN models, which indicate that blockade of VEGFA‐induced angiogenesis could be beneficial for patients suffering from DN.^21^, ^22^ In an attempt to further elucidate the underlying molecular mechanisms of DN, particularly the role of VEGFA in DN, five miRNA profiling tools were used to predict the potential target miRNAs of VEGFA, which were DIANA (http://diana.imis.athena-innovation.gr/DianaTools/index.php?r=microT_CDS/index), miRDB (http://www.mirdb.org/), TargetScan (http://www.targetscan.org/vert_71/), miRWalk (http://mirwalk.umm.uni-heidelberg.de/) and miRSearch (http://www.exiqon.com/microrna-target-prediction). Subsequently, Jvenn (http://jvenn.toulouse.inra.fr/app/example.html) was used to compare the predictions and screen the regulatory miRNAs.

### 2.3 Dual‐luciferase reporter gene assay

The bioinformatics prediction website (microRNA.org) was employed to determine the target gene of miR‐16‐5p, which indicated that miR‐16‐5p could bind to the 3′ untranslated region (3′UTR) of VEGFA. A dual‐luciferase reported gene assay was employed to further assess whether VEGFA was indeed a direct target gene of miR‐16‐5p. Next, miR‐16‐5p was co‐transfected with reporter plasmids pMIR‐VEGFA‐wild‐type (WT) or pMIR‐VEGFA‐mutant (MUT), respectively, into HEK‐293T cells (CRL‐1415, Shanghai Xinyu Biological Technology Co, Ltd). After a 48‐hours period of transfection, the cells were collected, lysed and centrifuged with the supernatant collected. A luciferase assay kit (RG005, Beyotime Biotechnology Co, Ltd) was then used to dissolve the Renilla luciferase detection buffer and firefly luciferase detection reagent. The buffer (100 μL/sample) was added with substrate (1:100) to prepare the Renilla luciferase detection working solution. A fluorometer was used to determine the luciferase activity. In each group, 20‐100 μL sample were added with 100 μL firefly luciferase detection reagent and 100 μL luciferase detection working solution, respectively, and fully mixed by means of pipetting followed by determination of the relative light unit (RLU). The reporter gene cell lysate was regarded as the blank control and renilla luciferase as an internal reference. Luciferase activity = RLU (firefly luciferase)/RLU (Renilla luciferase).

### 2.4 In vitro HG‐treated human podocytes (HPDCs) cultures

After the cryopreserved HPDCs had been thawed and centrifuged, the supernatant was removed and the cells at the bottom were re‐suspended with HPDC medium. The cells were then cultured in a 100‐cm^2^ plate with 8‐10 mL HPDC medium in the incubator (33°C, 5% CO_2_). When HPDCs had reached 70%‐80% confluence, they were treated with 0.05% trypsin solution at 37°C for 3 minutes, centrifuged at 1120 *g* for 5 minutes and re‐suspended in the HPDC medium. The cell suspension was then adjusted to a certain concentration and sub‐planted into 2‐3 culture plates (100 cm^2^), which were then cultured in a 33°C incubator or a 37°C incubator (both containing 5% CO_2_), respectively, to proliferate and differentiate. After 8 days of culturing, mature HPDCs were incubated in eluent for 12 hours and then classified into three groups: control (normal HPDCs), MA (HPDCs treated with 25 mmol/L isotonic mannitol) and HG (HPDCs treated with 25 mmol/L HG for 24 hours).

### 2.5 Cell treatment

The cells were transfected based on the instructions of the lipofectamine 2000 (11668‐019, Invitrogen Inc). In order to investigate the effects of miR‐16‐5p on HG‐treated HPDCs, HG‐treated HPDCs were treated with inhibited miR‐16‐5p or VEGFA and overexpressed miR‐16‐5p, and grouped into mimic‐negative control (NC) (HPDCs transfected with miR‐16‐5p mimic NC plasmid), miR‐16‐5p mimic (HPDCs transfected with miR‐16‐5p mimic), inhibitor NC (HPDCs transfected with miR‐16‐5p inhibitor NC plasmid), miR‐16‐5p inhibitor (HPDCs transfected with miR‐16‐5p inhibitor), miR‐16‐5p inhibitor + siRNA‐VEGFA (HPDCs transfected with miR‐16‐5p inhibitor and siRNA‐VEGFA plasmid), siRNA‐NC (HPDCs transfected with siRNA‐NC plasmid) and siRNA‐VEGFA (HPDCs transfected with siRNA‐VEGFA plasmid) groups. Then, the miR‐16‐5p in hUSCs were transferred to HG‐treated HPDCs via exosomes. Lentivirus was used to infect hUSCs. HEK‐293T cells were collected for transient cotransfection system. Subsequently, 1 μg PMD2G, 3 μg PSPAX2 and 4 μg Prutou3‐mChely/miR‐16‐5p (miR‐NC/miR‐16‐5p) were mixed and subjected to lentivirus packaging in a culture plate with a size of 60 mm. After a 24‐hours period of infection, the supernatant was collected. After the culture medium was replaced for another 24‐hours culturing, the supernatant was collected again. After the hUSCs were infected with the mixture of the two aforementioned supernatants, the lentiviral‐transduced hUSCs were seeded into a 24‐well plate at a density of 5 × 10^4^ cells/well and cultured overnight before transduction. The medium was replaced with a infection complex medium which was composed of 500 μL lentivirus supernatant, 500 μL fresh medium and 8 μg polyacrylamide (Sigma‐Aldrich Chemical Company) for the uptake of virus particles. A total of 6 groups were prepared as follows: HPDC (HPDCs without the addition of hUSCs), Exo‐depl (hUSCs co‐cultured with HPDCs in an exosome‐depleted medium), hUSCs miR‐16‐5p (hUSCs transfected with overexpressed miR‐16‐5p and co‐cultured with HPDCs), hUSCs miR‐16‐5p‐KD (hUSCs transfected with miR‐16‐5p inhibitor and co‐cultured with HPDCs), miR‐16‐5p mimic (HPDCs transfected with miR‐16‐5p mimic plasmid without hUSCs) and hUSCs miR‐16‐5p‐KD + VEGF‐KD (hUSCs transfected with miR‐16‐5p inhibitor and co‐cultured with HPDCs transfected with siRNA‐VEGFA plasmid).

### 2.6 Cell counting kit‐8 (CCK‐8) assay

Human podocytes in the logarithmic growth were collected and inoculated into a 96‐well plate and incubated in an incubator (37°C, 5% CO_2_). After culturing for 24 hours, the corresponding reagents were added into each group (each group contained 6 duplicated wells). After 24 hours of incubation, the CCK‐8 reagent (10 μL/well) was added into the 96‐well plate under aseptic conditions, after which the cells were cultured in the incubator for another 1.5 hours. The optical density (OD) values of each group were determined using an automatic microplate reader (Molecular Devices Corporation) at a wavelength of 490 nm. The measurement was repeated at least three times after which a mean OD value was obtained.

### 2.7 hUSCs culture and screening

Fresh midstream urine (200 mL) from healthy male adults was collected and added with 5 mL Penicillin‐Streptomycin (PS). The urine samples were then promptly sub‐packed into 4 × 50 mL sterile centrifuge tubes and centrifuged. After that, 20 mL phosphate buffer saline (PBS) containing 1% PS was added into each tube with 6 mL LMM101 medium added for re‐suspension purposes. The cell suspension was then transferred to 0.1% gelatin‐coated 6‐well plates and incubated at 37°C with 5% CO_2_. After 5‐7 days, the cell adherence was observed, and the LMM101 medium was replaced to remove non‐adherent cells. Finally, when the attached cells had reached 80% confluence, they were trypsinized and sub‐cultured.

### 2.8 Flow cytometry

The hUSCs at passage 3 were treated with 0.25% trypsin for 3 minutes, re‐suspended with LMM101 medium and centrifuged accordingly. The cell concentration was adjusted to 1 × 10^6^ cells/mL. Every 200‐μL cell suspension was packed into an Eppendorf (EP) Tube, respectively, and incubated with 5 μL different fluorescence‐labelled monoclonal antibodies (CD13, CD14, CD19, CD29, CD31, CD34, CD44, CD45, CD73, CD90, CD105, CD146, SSEA4, TRA‐1‐81, HLA‐ABC and HLA‐DR) for 15 minutes at 4°C under conditions void of light. Following that, 2 mL of PBS was added to each tube, centrifuged at 1120 *g* for 5 minutes and added with 400 μL PBS (0.01 mol/L) containing 0.5% paraformaldehyde. Immunoglobulin G (IgG) antibody labelled with fluorescence with the same colour served as the control group. Finally, the tubes were subjected to flow cytometry detection.

### 2.9 Osteoblasts and adipocytes differentiated from hUSCs

Directional differentiation of the hUSCs into osteoblasts was performed as follows: a total of 5 × 10^5^ hUSCs at passage 3 were inoculated in 24‐well plates. After the cell confluence reached more than 80%, 2 mL osteogenic inducing fluid (Gibco Company) or 2 mL adipogenic inducing fluid (Gibco Company) was added into each well. Cells in the control group were added with 2 mL LMM101 medium, with the medium changed at the regular intervals every 3 days. The inducing fluid was discarded after 21 days, after which the cells were fixed with 4% paraformaldehyde for 15 minutes at room temperature. The hUSCs treated with osteogenic inducing fluid were stained with 0.1% alizarin red aqueous solution (Sigma‐Aldrich Chemical Company) for 30 minutes. The hUSCs treated with adipogenic inducing fluid were stained with Oil red O solution (3:2; Sigma‐Aldrich Chemical Company) for 30 minutes after rinsed with 60% isopropanol. The results were observed and photographed under an optical microscope (Olympus Optical Co, Ltd).

### 2.10 Exo quick‐TC method

The hUSCs were collected 48 hours after lentiviruses infection, centrifuged at 28000 *g* for 15 minutes and added with Exo Quick‐TC exosome sediment (every 1 mL Exo Quick‐TC was added to 5 mL medium) at 4°C overnight. The Exo Quick‐TC/body fluid mixture was centrifuged at 17920 *g* for 30 minutes. Exosome sediment located at the bottom of the tube was observed to be light brown or white in colour.

### 2.11 Transmission electron microscopy (TEM)

The prepared exosomes were immediately fixed in 4% glutaraldehyde for at 4°C for 2 hours, washed three times with 0.1 mol/L PBS and then fixed with 1% osmium tetroxide for 2 hours. Semi‐thin slices at a thickness of 0.5 μm were prepared and positioned under a light microscope. Then, ultra‐thin slices with a thickness of 60 nm were prepared, stained with uranium acetate and lead citrate, and observed under a TEM. TEM was also used to observe the ultrastructure of podocytes in kidney tissues after the model was established. The podocyte, the average of foot process width (FPW) and the podocyte fusion rate were then calculated. (a) Podocytes: 3 glomeruli were observed in each case with 10 visual fields randomly selected to determine the number of podocytes; (b) FPW: the distance between the bilateral membrane of the foot process on the level of the pore membrane was measured in order to obtain the average value; (c) Podocyte fusion rate: the total length of the basement membrane was measured and regarded as X, then, after which the total length of the foot process fusion on the basement membrane was measured and regarded as Y. Finally, the fusion rate was calculated based on Y/X.

### 2.12 Co‐culture of HPDCs and hUSCs

Human podocytes and hUSCs were spread into a co‐culture chamber (diameter: 0.4 μm) at a ratio of 3:1, respectively. HPDCs (about 1.2 × 10^5^ cells) were placed into the basolateral chamber while the hUSCs (about 0.4 × 10^5^ cells) were placed into the apical chamber. The co‐culture chamber was placed in a 6‐well plate. During the co‐culturing process, the apical chamber was treated with Dulbecco's Modified Eagle Medium (DMEM) containing 10% serum, and the basolateral chamber was treated with 10% exosome‐free foetal bovine serum (FBS) medium. The cells were co‐cultured for 4‐5 days, with the medium changed every 1‐2 days.

### 2.13 Confocal microscopy observation

The carboxyfluorescein diacetate succinimidyl ester (CFSE) was diluted at a ratio of 1:1000 and then mixed with the exosomes suspension secreted from 20 μg of lentivirus‐infected hUSCs. After incubated at 37°C for 15 minutes, the exosomes labelled by CFSE were co‐cultured with HPDCs. The uptake of exosomes by HPDCs was observed under a confocal fluorescence microscopy at 12 hours, 24 hours and 48 hours during co‐culturing, respectively.

### 2.14 Reverse transcription‐quantitative polymerase chain reaction (RT‐qPCR)

A TRIzol kit (Invitrogen) was applied to extract the total RNA from cells in each group. Next, 1 μg total RNA was reversely transcribed into complementary DNA (cDNA) using PrimeScript^TM^ RT reagent kit along with gDNA Eraser kit (Takara). Real‐time PCR was performed using the SYBR^®^ Premix Ex Taq^TM^ (Tli RNase H Plus) kit (Takara) on the PCR instrument (ABI7500, Thermo). The reaction conditions were comprised of pre‐denaturation at 95°C for 10 minutes, 40 cycles of denaturation at 95°C for 15 seconds and annealing at 60°C for 30 seconds. U6 was served as the internal reference for miR‐16‐5p, and glyceraldehyde‐3‐phosphate dehydrogenase (GAPDH) was considered as the internal reference for VEGFA and nephrin. Then, 2^−ΔΔCt^ was considered to be a reflection of the ratio of the expression of the target gene in the experimental group and the control group. All the primers were provided by Shanghai GenePharma Co Ltd (Table 1).

**Table 1:** Primer sequences for RT‐qPCR

Gene	Primer sequence
miR‐16‐5p	F: 5′‐ ATAGAATCCTTGTATTATTATGTTTGGAC‐3′
	R: 5′‐ ATAGGATCCAAATTATACTAGCAGGA‐3′
U6	F: 5′‐ ATGACGTCTGCCTTGGAGAAC‐3′
	R: 5′‐ TC AGTGTGCTACGGAGTTCAG‐3′
VEGFA	F: 5′‐ GGGCTGCTGCAATGATGAA‐3′
	R: 5′‐ TCCGCATGATCTGCATAGTGA‐3′
nephrin	F: 5′‐ AAAGGTACCAGATAAGCAGGCAGCAGGAGT‐3′
	R: 5′‐ AAACTCGAGTTGCACACCTGGCTTCGGCCT‐3′
GAPDH	F: 5′‐ AAGCTGGTCATCAATGGGAAAC‐3′
	R: 5′‐ ACCCCATTTGATGTTAGCGG‐3′

Abbreviations: GAPDH, glyceraldehyde‐3‐phosphate dehydrogenase; miR‐16‐5p, microRNA‐16‐5p; RT‐qPCR, reverse transcription‐quantitative polymerase chain reaction; VEGFA, vascular endothelial growth factor A.

### 2.15 Western blot analysis

Total protein was extracted from cells in each group. Total protein (30 μg) were electrophoresed with polyacrylamide gel (PAGE). After electrophoresis, the proteins were transferred onto a polyvinylidene fluoride (PVDF) membrane (Amersham, NJ, USA) and blocked with 5% skimmed milk powder for 1 hour at room temperature. The membrane was then incubated with the primary antibodies of VEGFA (1 μg/mL, ab46154), nephrin (1:1000, ab216341), α‐smooth muscle actin (α‐SMA) (1:10 000, ab124964), Bcl‐2‐associated X protein (Bax) (1:1000, ab32503), caspase‐3 (1:500, ab13847), monocyte chemoattractant protein‐1 (MCP‐1) (1:2000, ab25124), transforming growth factor‐β1 (TGF‐β1) (2 μg/mL, ab92486), tumour necrosis factor‐α (TNF‐α) (1:2000, ab220210), CD9 (1:2000, ab92726), CD63 (1:2000, ab108950), CD81 (1:1000, ab109201), TSG101 (1:2000, ab125011), HSP90B1 (1:2000, ab3674) and GAPDH (1:10 000, ab181602) overnight (all antibodies were purchased from Abcam Inc). After washed three times with phosphate buffer saline Tween‐20 (PBST) for 10 minutes each time, the membrane was then incubated with horseradish peroxidase (HRP)‐labelled goat anti‐rabbit secondary antibody (1:10 000, Jackson Immunolabs) for 1 hour at room temperature, followed by 3 PBST washes, 10 minutes each time. The grey‐value analysis of protein bands was performed using Image‐Pro Plus 6.0 (Media Cybernetics) for determining relative protein level.

### 2.16 Terminal deoxynucleotidyl transferase (TdT)‐mediated 2′‐Deoxyuridine 5′‐Triphosphate (dUTP) nick‐end labelling (TUNEL)

The 24‐well plates with hUSCs were washed three times with PBS, fixed for 30 minutes with 4% paraformaldehyde and rinsed three times with PBS again. Then, the 24‐well plates with hUSCs were fixed by 0.3% H_2_O_2_‐formaldehyde solution (30% H_2_O_2_: formaldehyde = 1:99) for 30 minutes and treated with 0.3% Triton X‐100 for 2 minutes on ice. TUNEL reaction mixture was prepared according to TUNEL cell apoptosis detection kit (green fluorescence, C1088). Each experimental group was treated with 50 μL TdT and 450 μL fluorescein‐labelled dUTP solutions, while the NC group was added with 50 μL fluorescein‐labelled dUTP solution alone. After incubated at 37°C for 60 minutes under conditions void of light, the cells were then counterstained with diamidino‐phenyl‐indole (DAPI), mounted by anti‐fluorescence quenching mounting medium and observed under a fluorescence microscope.

### 2.17 Establishment of a rat model with diabetes

Fifty male Sprague Dawley (SD) rats (weighting: 180‐200 g) were adaptively fed for a week. The rats were fasted yet with free access to water for 12 hours prior to the model establishment. A total of 40 rats were randomly selected for modelling and injected intraperitoneally with 1% STZ solution (Thermo Fisher Scientific) at a dose of 65 mg/kg. The remaining 10 rats were injected with an equal volume of citric acid buffer as the normal group. As four rats died during model establishment, the successful rate was 90% (36/40). The blood glucose was determined for 3 consecutive days in a random fashion 72 hours after intraperitoneal injection with STZ solution. In the event, the random blood glucose was over 16.7 mol/L, and the 24 hours urine volume had doubled, the diabetes rat model was considered to be successfully established.^23^

### 2.18 Animal grouping and treatment

In addition to the normal group, the successfully established diabetic rats were randomly classified into three groups (at least 10 rats in each group): diabetes, diabetes + hUSCs‐Exo control and diabetes + hUSCs‐Exo miR‐16‐5p. Rats in the diabetes + hUSCs‐Exo control and diabetes + hUSCs‐Exo miR‐16‐5p groups were injected with 200 μL PBS containing 100 μg (protein content) exosomes (extracted from hUSCs infected with overexpressed miR‐16‐5p) and corresponding NC lentiviruses via tail vein, while rats in the diabetes group were injected with 200 μL pure PBS via tail vein. The above injections were given one time a week for consecutive 12 weeks. After that, metabolic cage was used to collect the urine at the 24th hours for determining urine protein (Upro) and urine creatinine (Ucr), and the rats in these four groups were euthanized. The blood glucose (Glu), kidney weight (KW) and body weight (BW) were measured, and an automatic biochemical analyzer (Hitachi) was used to detect renal functions, including that of blood urea nitrogen (BUN), serum creatinine (Scr) and creatinine clearance rate (CCR).

### 2.19 Haematoxylin‐eosin (HE) staining

After the collection of kidney tissues, routine fixation, dehydration, embedding, sectioning, staining and mounting were performed, after which the morphological changes of the podocytes were observed under an optical microscope (200×). A total of three visual fields were randomly selected for photographing. Afterwards, the surface area of renal tissue cells of the rats in each group was determined through the application of the automatic morphometery (HPIAS21000). The measurements were repeated five times in order to obtain average values.

### 2.20 Periodic acid‐Schiff (PAS) staining

After dewaxing and hydration, the paraffin slices were oxidized in periodic acid solution and stained with Schiff reagent (Beijing Leagene Biotech Co Ltd) for 30 minutes. The slices were then stained with haematoxylin for 5 minutes, dehydrated with 95% ethanol and absolute ethanol, cleared in xylene and mounted with neutral gum. The hyperplasia area of the purple‐red mesangial matrix of each rat was calculated using Image‐Pro Plus software (Media Cybernetics Company), followed by observation and photography under an optical microscope. PAS staining results revealed the nucleus was blue, while the mesangial matrix, amyloid substance and fibrae sanguis were purple‐red.

### 2.21 TUNEL staining

The dewaxed slices were collected and incubated with 1% protease K diluent (50 μL) for 30 minutes at 37°C, followed by the addition of 0.3% H_2_O_2_ methanol solution to eliminate endogenous peroxidase (POD) activity. Next, the slices were added with 50 μL converter‐POD and developed by 2% diaminobenzidine (DAB). After the cells were observed to have a brown‐yellow nucleus under a microscope, the distilled water was added to terminate the reaction. Then, the slices were counterstained with haematoxylin, dehydrated by gradient alcohol, cleared, mounted and observed under an optical microscope (×400). Ten visual fields were randomly selected, after which the number of positive cells and podocytes were counted. The apoptotic positive cells had a brown‐yellow nucleus, while normal cells presented blue nucleus. After the average values had been obtained, the apoptotic index (AI) of podocytes was expressed as a ratio of the number of brown‐yellow cells to that of blue cells.

### 2.22 Statistical analysis

All data were analysed using SPSS 21.0 software (IBM) with equality of variances assessed. Normal distribution was tested for all the data by using D'Agostino and Pearson omnibus normality test. Measurement data subjected to normal distribution were expressed as mean ± standard deviation. Comparisons of mean values between two groups were analysed using an independent sample *t* test, while comparisons among multiple groups were analysed using one‐way analysis of variance (ANOVA). The post hoc test was applied for pairwise comparison of multiple groups with homogeneity of variance. Rank‐sum tests were used to compare the data failed to conform to normal distribution. A *P* value < .05 was considered to be significantly different.

## 3 RESULTS

### 3.1 miR‐16‐5p inhibits VEGFA expression

**Figure 1:**
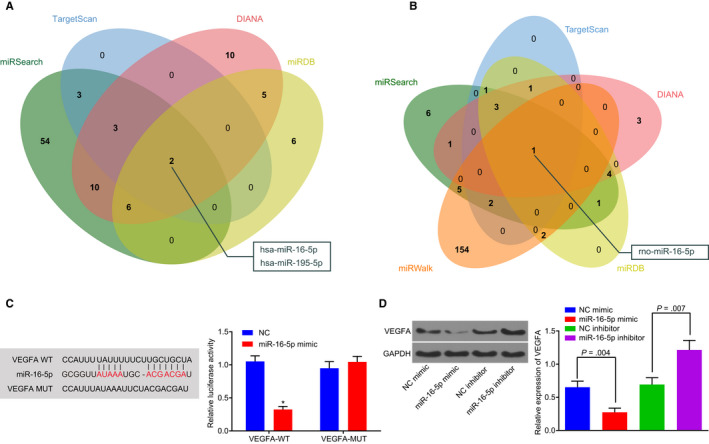
miR‐16‐5p targets VEGFA and inhibits its expression. (A,B) the miRNAs that could potentially be involved in the regulation of human or rat VEGFA predicted by miRNA‐gene relationship prediction websites, including DIANA, miRDB, TargetScan, miRWalk and miRSearch websites. The comparison of the predicted results was displayed by a Venn diagram. C, the targeting relationship between miR‐16‐5p and VEGFA predicted by dual‐luciferase reporter gene assay. D, Western blot analysis was performed to determine VEGFA expression in HPDCs stimulated by high glucose in the presence of miR‐16‐5p compared with that of miR‐16‐5p inhibitor. *P < .05 vs the VEGFA‐MUT group. Measurement data were expressed as mean ± standard deviation. Data between two groups were analysed using *t* test, and the experiment was repeated three times. HPDCs, human podocytes; miR‐16‐5p, microRNA‐16‐5p; MUT, mutant; VEGFA, vascular endothelial growth factor A

Based on the prediction data obtained from the DIANA, miRDB, TargetScan and miRSearch, 36, 19, 8 and 78 miRNAs, which were potentially involved in regulating human VEGFA, were obtained,. The comparisons of the predicted results were illustrated using a Venn diagram, which identified two miRNAs, has‐miR‐16‐5p and has‐miR‐195‐5p in the intersection (Figure 1A). Next, DIANA, miRDB, TargetScan, miRWalk and miRSearch were further used to predict the possible miRNAs that could regulate rat VEGFA. As depicted in Figure 1B, a total of 13 miRNAs with miTG score > 0.95 were screened out from DIANA, while 13 miRNAs with Target Score > 95 were found from miRDB. Moreover, 8 miRNAs with weighted context + + score ≤ −0.5 were detected in TargetScan, while 164 miRNAs with energy ≤ −20 and binding sites at 3′UTR were located in miRWalk. Additionally, 24 miRNAs were predicted to regulate VEGFA in miRSearch. Notably, there was only one miR, rno‐miR‐16‐5p, in the intersection based on the prediction results from these five databases (Figure 1B). Furthermore, has‐miR‐16‐5p and rno‐miR‐16‐5p were found to contain the same sequences (uagcagcacguaaauauuggcg) according to data obtained from the miRBase database (http://www.mirbase.org/) and the same binding sites in both miR‐16‐5p and VEGFA based on the data from the TargetScan website, indicating that miR‐16‐5p could bind to VEGFA.

In order to further verify the aforementioned results, a dual‐luciferase reporter gene assay was performed. Compared with the NC group, the activity of luciferase in the VEGFA‐WT group was significantly decreased in the presence of miR‐16‐5p mimic (*P* < .05), while no significant effect on the activity of luciferase in the VEGFA‐MUT group was observed (*P* > .05) (Figure 1C). HPDCs stimulated by HG were treated with overexpressed or inhibited miR‐16‐5p plasmids. Western blot analysis revealed that VEGFA expression in the miR‐16‐5p mimic group was significantly decreased compared with the mimic NC group (all *P* < .05). On the contrary, VEGFA expression in the miR‐16‐5p inhibitor group was significantly higher than that in the inhibitor NC group (all *P* < .05) (Figure 1D). Taken together, the above results demonstrate that miR‐16‐5p can specifically bind to VEGFA‐3′‐UTR and down‐regulate the expression of VEGFA.

### 3.2 HG‐stimulated HDPCs inhibits the expression of miR‐16‐5p and nephrin but promotes VEGFA expression

**Figure 2:**
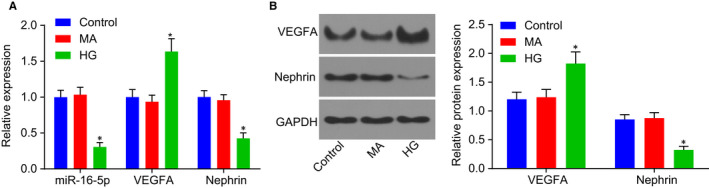
High glucose stimulation inhibits the expression of miR‐16‐5p and nephrin in HPDCs, whereas promotes the expression of VEGFA. A, RT‐qPCR was employed to determine the expression of miR‐16‐5p, VEGFA and nephrin in the control, MA and HG groups. B, Western blot analysis was used to determine the protein expression of VEGFA and nephrin in each group. *P < .05 vs the control group. Measurement data were expressed as mean ± standard deviation. Multivariate analysis of variance was used for comparison among multiple groups, and the experiment was repeated 3 times. HG, high glucose; HPDCs, human podocytes; miR‐16‐5p, microRNA‐16‐5p; RT‐qPCR, reverse transcription‐quantitative polymerase chain reaction; VEGFA, vascular endothelial growth factor A

In order to examine in detail the expression of miR‐16‐5p and VEGFA in HG‐stimulated podocytes, the cultured HPDCs were assigned into blank control (normal HPDCs), MA (HPDCs treated with 25 mmol/L isotonic mannitol) as a NC and HG (HPDCs treated with 25 mmol/L HG for 24 hours) groups. Next, RT‐qPCR and Western blot analysis were conducted to determine the expression of miR‐16‐5p, VEGFA and podocyte marker nephrin. As illustrated in Figure 2A‐B, there was no significant difference in the mRNA and protein level of VEGFA and nephrin, or the miR‐16‐5p expression in the MA group compared with the control group (all *P* > .05). Notably, the expression of miR‐16‐5p and nephrin was significantly decreased, whereas the expression of VEGFA was markedly elevated in the HG group compared with the control group and the MA group (all *P* < .05). The above results demonstrate that HG stimulation in HPDCs can inhibit the expression of miR‐16‐5p and nephrin while promoting the expression of VEGFA.

### 3.3 hUSCs have specific molecular markers of MSCs and the ability of multidirectional differentiation

**Figure 3:**
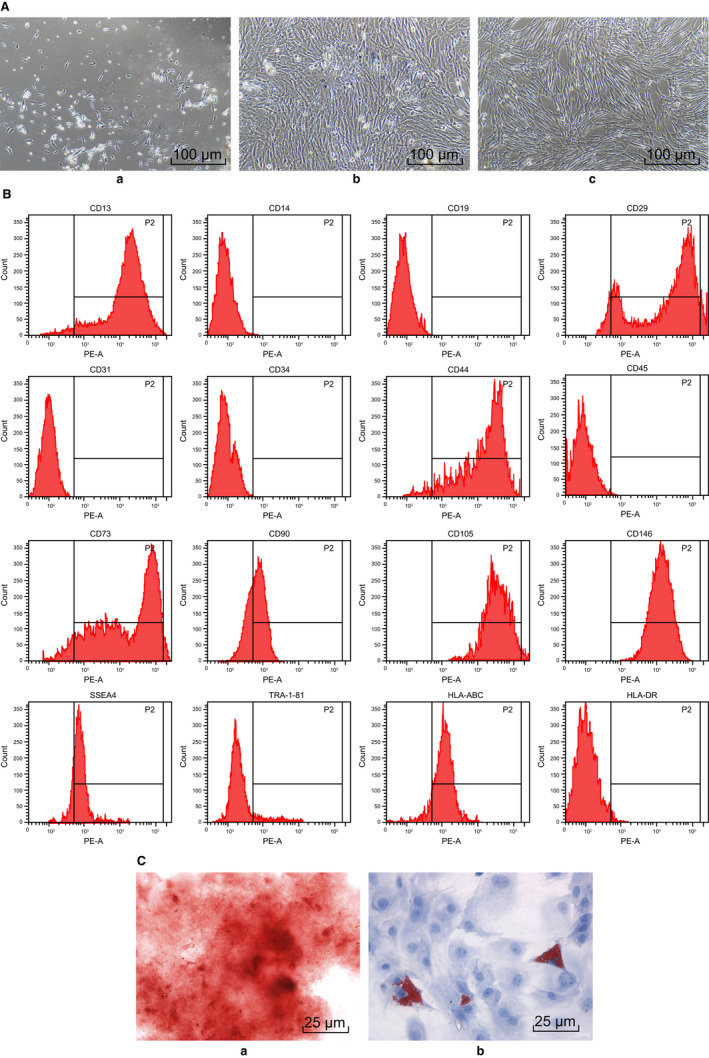
Human urine‐derived stem cells have specific molecular markers of MSCs and the ability of multidirectional differentiation. A, morphological observation of hUSCs (scale bar = 25 μm); a, 5 d after inoculation; b, 16 d after culture; c, 2‐3 wk after subculture. B, surface marker molecules of hUSCs detected by flow cytometry. C, hUSC osteogenic and adipogenic induction; a, directional differentiation of hUSCs into osteoblasts; b, directional differentiation of hUSCs into adipocytes (scale bar = 25 μm). hUSCs, human urine‐derived stem cells; MSCs, mesenchymal stem cells

In order to assess whether hUSCs possessed the ability of multidirectional differentiation into osteoblasts or adipocytes, the cells were treated with osteogenic induction and adipogenic induction. The cellular morphology was analysed first. After inoculation for 5 days, hUSCs were noted to have adhered to the wall in cobblestone‐like shape (Figure 3A‐a). Subsequently, the cells exhibited a clone‐like growth and reached 80%‐90% confluence after 16 days (Figure 3A‐b). Next, 2‐3 weeks after subculture, hUSCs retained slender morphology, grew and proliferated rapidly (Figure 3A‐c). Then, the expression of hUSCs surface antigen was analysed by flow cytometry. As illustrated in Figure 3B, CD13 was 92.58%, CD29 was 98.67%, CD44 was 95.11%, CD73 was 99.43%, CD90 was 44.2%, CD105 was 91.74%, CD146 was 81.66%, SSEA4 was 49.61%, TRA‐1‐81 was 25.55%, and HLA‐ABC was 67.59%, while the remaining were less than 1%. The results were consistent with the biological characteristics of MSCs, suggesting that hUSCs have specific molecular markers of MSCs. Furthermore, 21 days after osteogenic induction, hUSCs were found to have overlapped and formed calcified nodules comprising of a small amount of mineral salt sediment, indicating that the hUSCs had the potential to differentiate into osteoblasts (Figure 3C‐a). Besides, 14 days after adipogenic induction, hUSCs were observed to have a lipid sediment inside, with the lipid droplets gradually becoming larger and exhibiting a string‐like structure, indicating that the hUSCs were capable of differentiating into adipocytes (Figure 3C‐b). Thus, based on the above results, we conclude that hUSCs have the specific molecular markers of MSCs and could differentiate into osteoblasts and adipocytes.

### 3.4 hUSCs inhibit VEGFA expression in HG‐induced podocytes through exosomes‐secreted miR‐16‐5p

**Figure 4:**
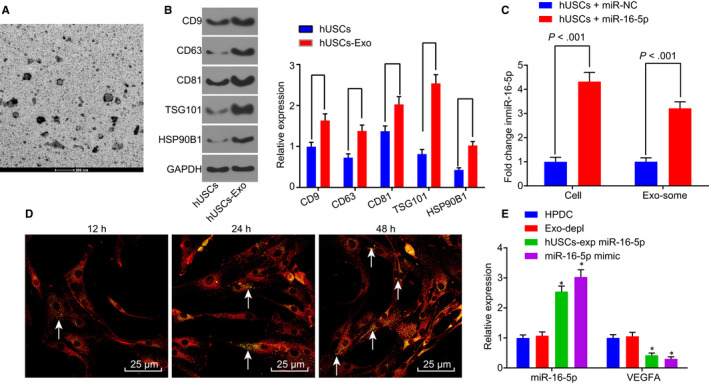
miR‐16‐5p is released to HPDCs via hUSC exosomes and effectively inhibits VEGFA in HPDCs. A, TEM was used to observe the morphology change in hUSCs‐Exo (scale bar = 200 nm). B, the expression of CD9, CD63, CD81, TSG101 and HSP90B1 was determined by Western blot analysis. C, RT‐qPCR was used to determine miR‐16‐5p expression in hUSCs and hUSC exosomes. D, the uptake of hUSC exosomes by HPDCs was observed at different time‐points (12 h, 24 h, and 48 h), white arrows pointed to the CFSE‐labelled exosomes (green), while HPDCs were red‐stained (scale bar = 25 μm). E, expression of miR‐16‐5p and VEGFA was determined in each group by RT‐qPCR. *P < .05 vs the HPDC group. Measurement data were expressed as mean ± standard deviation. Independent sample *t* test was used for comparison between two groups, and multivariate analysis of variance was employed in the comparison between multiple groups. The experiment was repeated three times. CFSE, carboxyfluorescein diacetate succinimidyl ester; Exo‐depl, hUSCs co‐cultured with HPDCs in a exosome‐depleted medium; HPDCs, human podocytes; hUSCs, human urine‐derived stem cells; miR‐16‐5p, microRNA‐16‐5p; RT‐qPCR, reverse transcription‐quantitative polymerase chain reaction; TEM, transmission electron microscopy; VEGFA, vascular endothelial growth factor A

The size of the vesicles in the hHUSCs‐Exo was varied when observed under a TEM, ranging from 30 nm to 120 nm (Figure 4A). The morphology was generally the same as the round or oval membranous vesicles. After staining, the complete envelope containing low‐density electron‐dense deposits was found in the vesicle. Western blot analysis was employed to further verify the presence of hHUSCs‐Exo and to detect the evolutionarily conserved proteins (CD9, CD63, CD81, TSG101 HSP90B1) as exosome surface markers. As depicted in Figure 4B, CD9, CD63, CD81, TSG101 and HSP90B1 were expressed in the exosomes derived from hUSCs, indicating the presence of hUSCs‐Exo in hUSCs.

In order to determine whether exogenous miR‐16‐5p could be transferred into HPDCs from hUSCs through exosomes, the expression of miR‐16‐5p in hUSCs and exosomes derived from hUSCs was determined by RT‐qPCR. As displayed in Figure 4C, compared with miR‐NC‐treated cells, the expression of miR‐16‐5p was significantly higher in hUSCs infected with lentiviruses of miR‐16‐5p and in exosomes derived from the hUSCs (all *P* < .05). These results suggested that hUSCs could effectively release exosomes enriched with miR‐16‐5p. In order to further elucidate that exosomes released by hUSCs could transfer miR‐16‐5p to HPDCs, exosomes traced by CFSE were co‐cultured with HPDCs. The uptake of exosomes by HPDCs was observed under a confocal fluorescence microscopy at 12, 24 and 48 hours after co‐culture (Figure 4D). Over the passage of co‐culture, more and more HPDCs presented green fluorescence, suggesting that the number of CFSE‐exosomes uptaken by HPDCs had increased in a progressive manner. After 48 hours of co‐culture, the uptake of CFSE‐exosomes by HPDCs was obvious, indicating that exosomes could be transferred from donor cells, hUSCs, to recipient cells, HPDCs.

In order to assess whether the in vitro transfer of miR‐16‐5p effectively inhibited endogenous VEGFA in HPDCs, the expression of miR‐16‐5p and VEGFA in HPDCs co‐cultured with hUSCs was determined by RT‐qPCR. Initially, whether exosomes were involved in the inhibition process was assessed, and the exosome‐depleted medium was used to inhibit exosome secretion. As shown in Figure 4E, there was no significant change regarding the expression of miR‐16‐5p and VEGFA in the Exo‐depl group compared with the HPDC group (*P* > .05). Interestingly, the expression of miR‐16‐5p was sharply elevated in the hUSCs‐Exo miR‐16‐5p as well as the miR‐16‐5p mimic groups (all *P* < .05), while in contrast, the expression of VEGFA was decreased (all *P* < .05). The results obtained indicated that the transfer of exogenous miR‐16‐5p from hUSCs to HPDCs is dependent on exosomes, and miR‐16‐5p through in vitro transfer could effectively inhibit VEGFA in HPDCs.

### 3.5 Secretion of miR‐16‐5p from hUSC exosomes can ameliorate podocytes injury induced by HG

**Figure 5:**
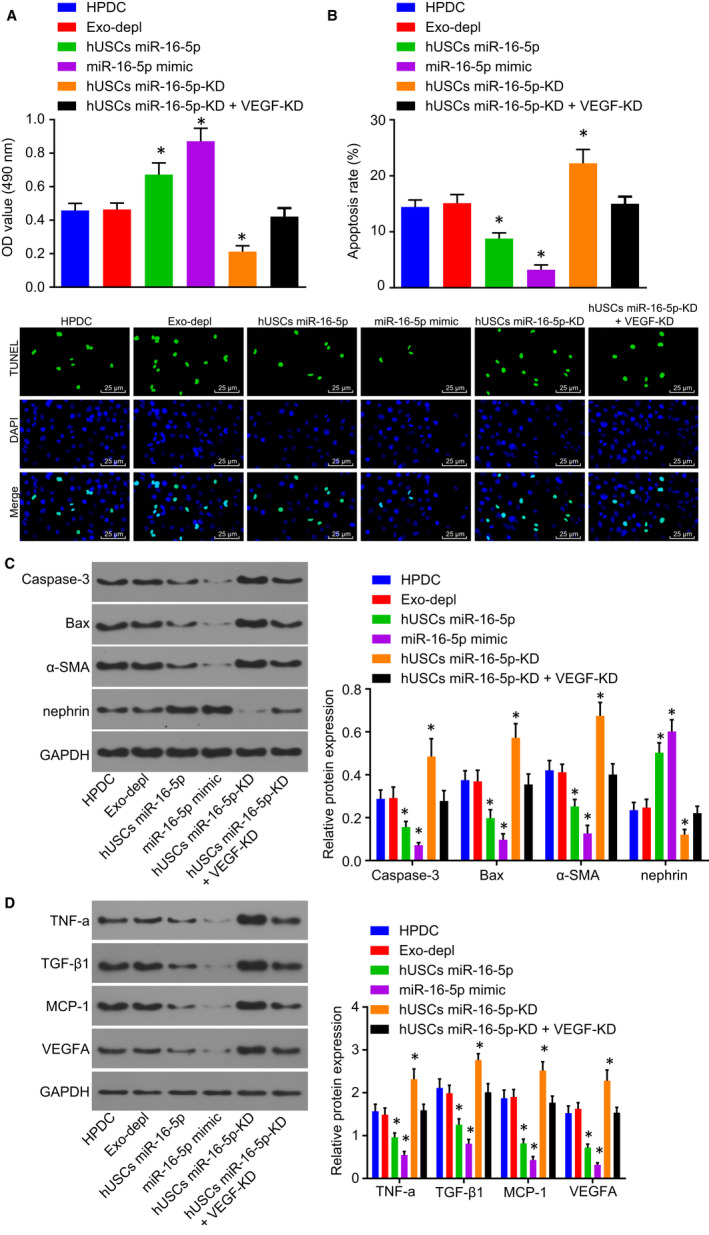
Secretion of miR‐16‐5p from hUSC exosomes ameliorates podocyte injury induced by HG. A, the viability of podocytes was detected by CCK‐8 assay. B, TUNEL was performed to measure the apoptosis rate of podocytes (scale bar = 25 μm). C, Western blot analysis was applied to determine the expression of nephrin, α‐SMA, Bax and Caspase‐3. D, the expression of VEGFA and relate factors MCP‐1, TGF‐β1 and TNF‐α examined by Western blot analysis. *P < .05 vs the HPDC group. Measurement data were expressed as mean ± standard deviation. Multivariate analysis of variance was used for comparison among multiple groups, and the experiment was repeated 3 times. CCK‐8, Cell Counting Kit‐8; Exo‐depl, hUSCs co‐cultured with HPDCs in a exosome‐depleted medium; HG, high glucose; HPDCs, human podocytes; hUSCs miR‐16‐5p‐KD + VEGF‐KD, hUSCs transfected with miR‐16‐5p inhibitor and siRNA‐VEGFA plasmid and co‐cultured with HPDCs; hUSCs miR‐16‐5p‐KD, hUSCs transfected with miR‐16‐5p inhibitor and co‐cultured with HPDCs; hUSCs, human urine‐derived stem cells; MCP‐1, monocyte chemoattractant protein‐1; miR‐16‐5p, microRNA‐16‐5p; TGF‐β1, transforming growth factor‐β1; TNF‐α, tumour necrosis factor‐α; TUNEL, Terminal deoxynucleotidyl transferase (TdT)‐mediated 2′‐Deoxyuridine 5′‐Triphosphate (dUTP) nick‐end labelling; VEGFA, vascular endothelial growth factor A

In order to investigate the effect of miR‐16‐5p secreted by hUSC exosomes on HG‐induced podocyte injury, CCK‐8 assay and TUNEL were applied to detect the podocyte viability and apoptosis rate after HG treatment. No significant difference was detected between the Exo‐depl and hUSCs miR‐16‐5p‐KD + VEGF‐KD groups as well as the HPDC group regarding the podocyte viability and apoptosis rate (*P* > .05) (Figure 5A‐B). However, the podocyte viability was promoted, while the apoptosis rate was decreased in the hUSCs miR‐16‐5p and miR‐16‐5p mimic groups remarkably (*P* < .05). In contrast, the hUSCs miR‐16‐5p‐KD group exhibited significantly repressed podocyte viability and increased rate of apoptosis (*P* < .05).

To further confirm miR‐16‐5p was correlated with podocyte protection, the protein expression of the podocyte surface marker protein (nephrin), MSC surface marker protein (α‐SMA) and apoptotic protein (Bax and Caspase‐3) was determined by means of Western blot analysis. The results obtained revealed there to be no significant difference in regard to the protein expression of nephrin, α‐SMA, Bax and Caspase‐3 in the Exo‐depl and hUSCs miR‐16‐5p‐KD + VEGF‐KD groups compared with the HPDC groups (*P* > .05) (Figure 5C). Notably, the protein expression of nephrin was up‐regulated, while that of Bax, Caspase‐3 and α‐SMA was down‐regulated in the podocytes of the hUSCs miR‐16‐5p and miR‐16‐5p mimic groups (all *P* < .05). On the contrary, the protein expression of nephrin was down‐regulated while that of Bax, Caspase‐3 and α‐SMA was up‐regulated in podocytes of the hUSCs miR‐16‐5p‐KD group (all *P* < .05).

Afterwards, Western blot analysis was applied to examine the protein levels of VEGFA and related factors MCP‐1, TGF‐β1 and TNF‐α in HPDCs, the results (Figure 5D) of which revealed that compared with the HPDC group, the expression of VEGFA, MCP‐1, TGF‐β1 and TNF‐α displayed no significant difference in the HPDCs of the Exo‐depl and hUSCs miR‐16‐5p‐KD + VEGF‐KD groups (*P* > .05), decreased in the HPDCs of the hUSCs miR‐16‐5p and miR‐16‐5p mimic groups (all *P* < .05), and increased in the HPDCs of the hUSCs miR‐16‐5p‐KD group (all *P* < .05).

Taken together, the aforementioned results indicated that the secretion of miR‐16‐5p from hUSC exosomes could ameliorate podocyte injury induced by HG.

### 3.6 Silencing of VEGA can attenuate HG‐induced podocyte injury

**Figure 6:**
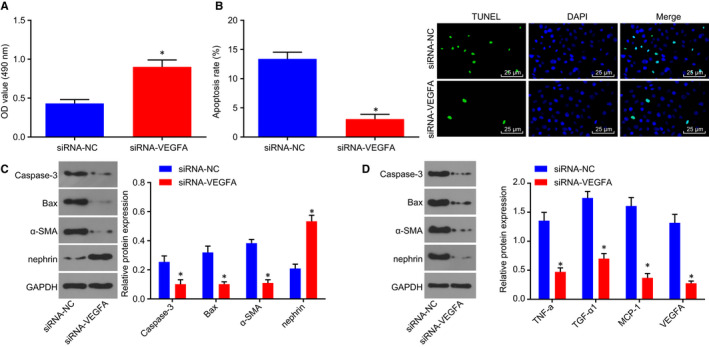
Depletion of VEGFA contributes to relieved podocyte injury induced by HG. The expression of VEGFA was depleted. A, the viability of podocytes was detected by CCK‐8 assay. B, TUNEL was performed to measure the apoptosis rate of podocytes (scale bar = 25 μm). C, Western blot analysis was applied to determine the expression of nephrin, α‐SMA, Bax and Caspase‐3. D, the expression of VEGFA and relate factors MCP‐1, TGF‐β1 and TNF‐α examined by Western blot analysis. Measurement data were expressed as mean ± standard deviation. Multivariate analysis of variance was used for comparison among multiple groups, and the experiment was repeated three times. *P < .05 vs the siRNA‐NC group. CCK‐8, Cell Counting Kit‐8; HG, high glucose; MCP‐1, monocyte chemoattractant protein‐1; TGF‐β1, transforming growth factor‐β1; TNF‐α, tumour necrosis factor‐α; TUNEL, Terminal deoxynucleotidyl transferase (TdT)‐mediated 2′‐Deoxyuridine 5′‐Triphosphate (dUTP) nick‐end labelling; VEGFA, vascular endothelial growth factor A

In order to detect the effect of VEGFA silencing on HG‐induced podocyte injury, CCK‐8 assay and TUNEL staining were conducted to detect the podocyte viability and apoptosis rate after VEGFA was depleted in hUSCs (Figure 6A‐B). The results showed that relative to the siRNA‐NC group, the podocyte viability was enhanced and the apoptosis rate was reduced in the siRNA‐VEGFA group (all *P* < .05).

Next, Western blot analysis was performed to determine the protein expression of nephrin, Bax, Caspase‐3, α‐SMA, VEGFA, MCP‐1, TGF‐β1 and TNF‐α in the podocytes (Figure 6C‐D). The results revealed that when compared with the siRNA‐NC group, the siRNA‐VEGFA group displayed increased protein expression of nephrin and decreased protein expression of Bax, Caspase‐3, α‐SMA, VEGFA, MCP‐1, TGF‐β1 and TNF‐α in the podocytes (all *P* < .05). Hence, depletion of VEGFA could relieve podocyte injury induced by HG.

### 3.7 Overexpressed miR‐16‐5p in hUSCs‐Exo protects the podocytes in diabetic rats

Next, in order to assess whether the protective effect of hUSCs‐Exo on nephrocytes exists in diabetic rats, DN rat models were established. After STZ injection for 72 hours, the random blood glucose of rats in the model group was examined at the same time‐point for three consecutive days, with the blood glucose detected to be higher than 16.7 mol/L (Table 2). Furthermore, in order to evaluate the protective effect of hUSCs‐Exo on nephrocytes in diabetic rats, hUSC exosomes containing overexpressed miR‐16‐5p were injected into the tail veins of the DN rats. The rats were then assigned into normal, diabetes, diabetes + hUSCs‐Exo control and diabetes + hUSCs‐Exo miR‐16‐5p groups. The Glu, KW, BW, BUN, Scr, CCR, Upro and Ucr of rats were measured, with the results collected demonstrating that compared with the normal group Glu, KW, BUN, Scr, CCR, Ucr and Upro were increased, while BW was obviously decreased in the other three groups (all *P* < .05). Interestingly, Glu, KW, BUN, Scr, CCR, Ucr and Upro exhibited marked reductions in the diabetes + hUSCs‐Exo miR‐16‐5p group compared with the diabetes + hUSCs‐Exo control group (*P* < .05), but no significant changes were observed in BW (*P* > .05) (Table 2).

**Table 2:** The effect of hUSCs on diabetic rats

Variable	Normal	Diabetes	Diabetes + hUSCs‐Exo control	Diabetes + hUSCs‐Exo miR‐16‐5p
Glu (mmol/L)	5.52 ± 0.47	25.45 ± 3.26^4^	20.53 ± 2.17*, #	16.34 ± 1.33*, #
KW (g)	1.35 ± 0.15	2.58 ± 0.37^4^	2.12 ± 0.27*, #	1.73 ± 0.16*, #
BW (g)	362.00 ± 15.35	242.54 ± 12.23^4^	251.85 ± 14.24^4^	253.45 ± 8.75^4^
BUN (mmol/L)	8.24 ± 0.78	18.72 ± 1.25^4^	15.35 ± 1.01*, #	12.01 ± 0.84*, #
Scr (μmol/L)	66.47 ± 5.72	99.27 ± 7.57^4^	87.68 ± 6.54*, #	78.23 ± 3.87*, #
CCR (mmol/L)	1.05 ± 0.14	2.25 ± 0.25^4^	1.70 ± 0.11*, #	1.41 ± 0.18*, #
Ucr (μmol/L)	89.46 ± 9.11	423.37 ± 45.48^4^	245.82 ± 27.48*, #	287.37 ± 24.99*, #
Upro (mg/24 h)	6.47 ± 0.77	35.57 ± 4.37^4^	14.85 ± 1.66*, #	19.37 ± 1.77*, #

Note

Measurement data were expressed as mean ± standard deviation, data among multiple groups were analysed using one‐way analysis of variance, and the experiment was repeated three times.

Abbreviations: BUN, blood urea nitrogen; BW, body weight; CCR, creatinine clearance rate; Glu, blood glucose; hUSCs, human urine‐derived stem cells; KW, kidney weight; miR‐16‐5p, microRNA‐16‐5p; Scr, serum creatinine; Ucr, urine creatinine urine protein; Upro, urine protein.

* *P* < .05 vs the normal group;

^#^ *P* < .05 vs the diabetes + hUSCs‐Exo control group.

**Figure 7:**
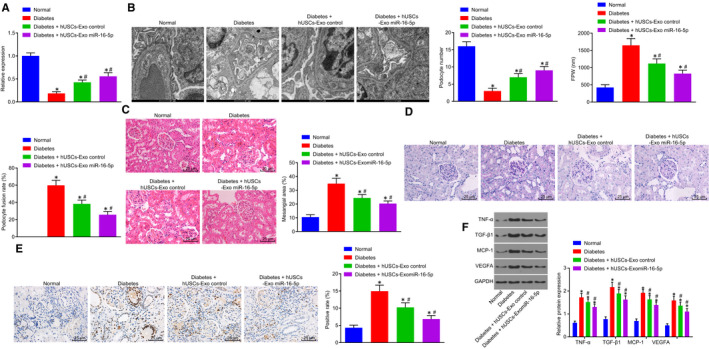
hUSC‐miR‐16‐5p overexpression has a protective effect on podocytes in diabetic rats. A, miR‐16‐5p expression was measured by RT‐qPCR. B, TEM was used to observe the ultrastructure of podocytes, to count podocytes and to calculate the average of FPW and podocyte fusion rate (scale bar = 1 μm). C, the pathological changes of kidney tissue detected by HE staining (scale bar = 25 μm). D, PAS staining was used to observe the pathological changes of kidney tissue (scale bar = 25 μm). E, TUNEL was used to detect the apoptosis rate of kidney tissue (scale bar = 25 μm). F, the expression of VEGFA, MCP‐1, TGF‐β1 and TNF‐α was determined by Western blot analysis. *P < .05 vs the normal group; #*P* < .05 vs the diabetes group. Measurement data were expressed as mean ± standard deviation. Data among multiple groups were analysed using one‐way analysis of variance, and the experiment was repeated three times. FPW, foot process width; HE, haematoxylin‐eosin; hUSCs, human urine‐derived stem cells; MCP‐1, monocyte chemoattractant protein‐1; miR‐16‐5p, microRNA‐16‐5p; PAS, periodic acid‐Schiff; RT‐qPCR, reverse transcription‐quantitative polymerase chain reaction; TEM, transmission electron microscopy; TGF‐β1, transforming growth factor‐β1; TNF‐α, tumour necrosis factor‐α; TUNEL, Terminal deoxynucleotidyl transferase (TdT)‐mediated 2′‐Deoxyuridine 5′‐Triphosphate (dUTP) nick‐end labelling; VEGFA, vascular endothelial growth factor A

Subsequently, RT‐qPCR methods were employed to determine the expression of miR‐16‐5p in kidney tissue. The obtained results revealed that the expression of miR‐16‐5p was reduced in the diabetes, diabetes + hUSCs‐Exo control and diabetes + hUSCs‐Exo miR‐16‐5p groups compared with the normal group (*P* < .05). However, when compared with the diabetes + hUSCs‐Exo control group, the expression of miR‐16‐5p was increased in the diabetes + hUSCs‐Exo miR‐16‐5p group (*P* < .05), while diminished levels were detected in the DN group (*P* < .05) (Figure 7A).

Furthermore, the morphology changes of podocytes in each group were observed by a TEM (Figure 7B). In the normal group, the podocyte structure was observed to be complete, with a neat and clear foot process arrangement, in addition to no observed thickening of the basement membrane. In the diabetes, diabetes + hUSCs‐Exo and diabetes + hUSCs‐Exo miR‐16‐5p groups, endothelial cell injury, basement membrane thickening and podocyte exfoliation and widened foot process space in addition to foot process fusion were detected. Remarkably, compared with the diabetes + hUSCs‐Exo control group, the injury of podocytes in the diabetes + hUSCs‐Exo miR‐16‐5p group was alleviated (all *P* < .05), and the injury of podocytes in the diabetes group was aggravated (all *P* < .05).

In order to further verify the protective effect associated with overexpressed miR‐16‐5p in hUSCs on rat nephrocytes, podocytes in each group were stained using HE, PAS and TUNEL. As illustrated in Figure 7C‐D, the glomeruli of the rats in the normal group were observed to be clear in structure, regular in shape, with the cells in the glomeruli regularly arranged with clear a basement membrane. However, the volume of glomerulus was increased, while the mesangial area was widened in addition to a greater matrix among the rats in the diabetes group. Moreover, the mesangial matrix hyperplasia of the rats in the diabetes + hUSCs‐Exo control and diabetes + hUSCs‐Exo miR‐16‐5p groups was more significantly ameliorated when compared to the diabetes group (*P* < .05). Compared with the diabetes + hUSCs‐Exo control group, the hyperplasia of mesangial matrix in the diabetes + hUSCs‐Exo miR‐16‐5p group was also alleviated (all *P* < .05). TUNEL assay results demonstrated that the apoptosis rate of nephrocytes in the diabetes + hUSCs‐Exo control group was remarkably higher than that in diabetes + hUSCs‐Exo miR‐16‐5p group (*P* < .05) (Figure 7E).

Changes to VEGFA and related factors MCP‐1, TGF‐β1 and TNF‐α in nephrocytes of diabetic rats were detected by Western blot analysis. The expression of VEGFA, MCP‐1, TGF‐β1 and TNF‐α was increased in the diabetes, diabetes + hUSCs‐Exo control and diabetes + hUSCs‐Exo miR‐16‐5p groups compared with the normal group (all *P* < .05) (Figure 7F). Meanwhile, compared with the diabetes + hUSCs‐Exo control group, the expression of VEGFA, MCP‐1, TGF‐β1 and TNF‐α was reduced in the diabetes + hUSCs‐Exo miR‐16‐5p group (all *P* < .05), while elevated levels were detected in the diabetes group (all *P* < .05). The aforementioned results indicate that overexpressed miR‐16‐5p in hUSCs confers a protective effect to the podocytes of diabetic rats.

## 4 DISCUSSION

Diabetic nephropathy commonly progressively manifests itself via haemodynamic and structural changes, which are often reflected by a decreased glomerular filtration rate and large amount of proteinuria.^22^ Studies have implicated hUSCs‐Exo in the process of wound repair in STZ‐induced diabetic mice.^24^ The key findings of the present study revealed that the hUSCs‐Exo overexpressing miR‐16‐5p could confer protection to HPDCs induced by HG, resulting in the suppression of VEGFA expression and podocyte apoptosis, and enhancement of podocyte proliferation.

Firstly, we found that miR‐16‐5p could regulate VEGFA, which was verified by the five predictive databases of miRNAs‐gene relationships and dual‐luciferase reporter gene assay. As a new class of transcription regulators, miRNAs have been shown to play important roles in hyperglycaemia‐induced podocyte dysfunction.^25^ Reports have previously demonstrated that the overexpression of miR‐29a ameliorates podocyte dysfunction induced by hyperglycaemia through the promotion of nephrin acetylation, highlighting its protective effect on diabetic podocytopathy.^26^ The in vitro and in vivo experiments results revealed that secretion of miR‐16‐5p from hUSC exosomes could ameliorate podocyte injury induced by HG. miR‐16‐5p is often considered as an endogenous control in tissues, serum and plasma, and acts as a tumour suppressor of gastric cancer.^27^ VEGFA is considered to be a driving factor associated with angiogenesis, migration, permeability and cell survival, with studies linking its abnormal expression in the kidney to a large array of renal diseases.^28^ A wide consensus exits regarding the notion that VEGF in DN has been shown to improve glomerular permeability and proteinuria.^21^ Elevated glomerular VEGFA expression in DN animal models have been attributed to the effect of HG on the production of VEGFA in podocytes, indicating that inhibition of VEGFA can be helpful in the treatment of renal complications.^17^ During the current study, evidence was obtained indicating that hUSCs overexpressing miR‐16‐5p led to a decrease in the levels of VEGFA, MCP‐1, TGF‐β1 and TNF‐α which were increased following the establishment of DN. Consistent with our findings, a previous study concluded that the up‐regulation of VEGFA in podocytes results in changes to glomerular selectivity and filtration, loss of podocytes and a decline in renal function in cases of DN.^29^ Accumulating studies have highlighted the regulatory effect of miRNAs on the expression of MCP‐1, TGF‐β1 and TNF‐α. For instance, overexpressed miR‐124a reduced the expression of MCP‐1, miR‐16 suppressed the epithelial‐mesenchymal transition (EMT) induced by TGF‐β1 in non–small‐cell lung carcinoma cells (NSCLC) and miR‐16 decreased the expression of TNF by Bcl‐2 in liver failure.^30^, ^31^, ^32^ Additionally, increased miR‐16‐1 and TNF‐α expression has been detected in the astrocytes of epilepsy mice,^33^ which although contradictory to the findings of our study, emphasized the regulatory role of miR‐16 and TNF‐α.

Mesenchymal stem cells regenerative therapy is a novel way of renal regeneration, where MSCs can repair the nephron through differentiation.^34^ Studies have indicated that MSC‐derived exosomes play a protective role in renal injury induced by ischaemia‐reperfusion.^35^ Interestingly, the paracrine mechanism, in which exosome is involved, has been shown to provide a therapeutic effect via stem cell transplantation.^36^ Bearing in mind the advantages of both hUSCs and exosomes, hUSCs‐Exo have been shown to be associated with a smaller rate of immune rejection, better differentiation, and more stable and sufficient supply, highlighting its potential role as a regenerative medicine treatment tool.^19^ Likewise, a previous study concluded that hUSCs‐Exo can alleviate renal damage in type I diabetic rats, while demonstrating the therapeutic role played by exosomes through the suppression of podocyte apoptosis as well as promotion of angiogenesis and cell survival.^19^

## 5 CONCLUSIONS

**Figure 8:**
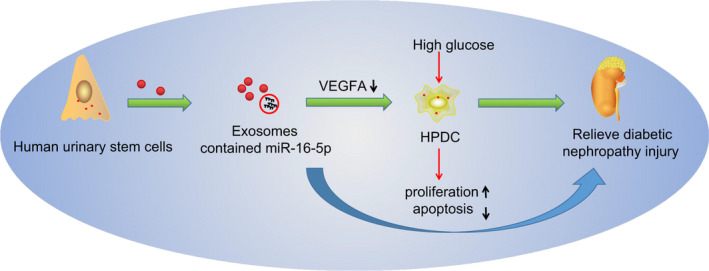
A schematic diagram depicts molecular basis underlying DN treatment by hUSCs. The secretion of hUSC exosomes promoted the podocyte proliferation and inhibited the podocyte apoptosis to protect against HG‐induced podocyte injury, thereby alleviating the damage of diabetic nephropathy by down‐regulating VEGFA *via* exosomes carrying overexpressed miR‐16‐5p. DN, diabetic nephropathy; HG, high glucose; hUSCs, human urine‐derived stem cells; miR‐16‐5p, microRNA‐16‐5p; VEGFA, vascular endothelial growth factor A

All data together allow us to propose a model of molecular mechanisms underlying DN treatment by hUSCs (Figure 8). hUSCs‐Exo overexpressing miR‐16‐5p could act to inhibit VEGFA while promoting proliferation and inhibiting the apoptosis of podocytes, all of which alleviate the damage inflicted by DN by protecting against podocyte injury. In conclusion, hUSCs‐Exo overexpressing miR‐16‐5p presents a fresh perspective, for which future therapeutic approaches for DN may be premised upon. However, as the research is still in the preclinical stage, further investigation into the finer mechanisms is needed in future studies.

## CONFLICT OF INTEREST

The authors have declared that no competing interests exist.

## AUTHOR CONTRIBUTIONS

Yu‐Rui Duan and Bao‐Ping Chen designed the study. Fang Chen, Su‐Xia Yang and Chao‐Yang Zhu collated the data, carried out data analyses and produced the initial draft of the manuscript. Ya‐Li Ma, Yang Li and Jun Shi contributed to drafting the manuscript. All authors have read and approved the final submitted manuscript.
